# Conditional transparency: Differentiated news framings of COVID-19 severity in the pre-crisis stage in China

**DOI:** 10.1371/journal.pone.0252062

**Published:** 2021-05-24

**Authors:** Yipeng Xi, Anfan Chen, Aaron Ng

**Affiliations:** 1 Department of Communications and New Media, National University of Singapore, Singapore, Singapore; 2 School of Humanity and Social Science, University of Science and Technology of China, Anhui Province, China; Neijiang Normal University, CHINA

## Abstract

Transparency of Chinese media coverage became an international controversy when the COVID-19 outbreak initially emerged in Wuhan, the eventual crisis epicenter in China. Unlike studies characterizing mass media in authoritarian contexts as government mouthpieces during a crisis, this study aims to disaggregate Chinese media practices to uncover differences in when, where, and how the severity of COVID-19 was reported. We examine differences in how media institutions reported the severity of the COVID-19 epidemic in China during the pre-crisis period from 1 January 2020 to 20 January 2020 in terms of both the “vertical” or hierarchical positions of media institutions in the Chinese media ecosystem and the “horizontal” positions of media institutions’ social proximity to Wuhan in terms of geographical human traffic flows. We find that the coverage of crisis severity is negatively associated with the media’s social proximity to Wuhan, but the effect varies depending on the positional prominence of a news article and situation severity. Implications of the institutions’ differentiated reporting strategies on future public health reporting in an authoritarian context are also discussed.

## Introduction

The first case of COVID-19 was discovered in Wuhan in December 2019 but Chinese news media did not officially acknowledge the seriousness of transmissibility until 20 January 2020. As a result, health information disclosure by Chinese media in the initial stage of the COVID-19 outbreak in China became an international point of contention [1]. The balance between information disclosure or control is often a dilemma for crisis managers, resulting in the sense of paralysis because a choice must be made between diametrically opposed options, each having considerable pros and cons [2]. Such longstanding tensions between control and free flow of information are also inherent in China’s authoritarian style of governance. While strong information control can facilitate longevity in governance through the reduction of public accountability, extreme information asymmetry between the governing and the governed is dangerous because it blinds the former to festering social problems and issues experienced by the latter until they erupt as protests [3, 4]. During a crisis, these tensions are even more pronounced.

Although authoritarian governments tend to exert strong control over access to crisis information through heavy-handed media management [5], tactics of “authoritarian resilience” and “creative adaptation” allow some degree of media independence in such a restrictive environment [6, 7]. Therefore, a confrontational or anti-state perspective may neglect the tensions within the authoritarian institutions and the governmental efforts to manage the state-citizen conflicts. Thus, rather than considering Chinese media as a monolithic entity at the beck and call of the government, this study considers Chinese media as a large ecosystem of different media institutions with diverse interests. Although Chinese media organizations are obliged to represent the party’s positions, protecting the reputation, legitimacy, and authority of the Chinese Communist Party (CCP) [8–10], they also have to adhere to professional news reporting ethos to timely and accurately report information that is in the public’s interest.

Building upon the theory of fragmented authoritarianism, we argue that Chinese power hierarchies within the Chinese media structure (i.e., institutions at different levels of the hierarchy have distinct degrees of power and autonomy) and social proximity to Wuhan generally moderated the severity framing of the epidemic in public health news reporting during the pre-crisis stage of the COVID-19 epidemic, but the level of moderation depends on the article’s placement prominence and the seriousness of evolving situations. Public health news reporting in an authoritarian Chinese context is much more complex than parroting government propaganda; it is a dynamic process resulting from the interactions between media institutions’ levels, social proximity, and strict media restrictions imposed by the government. In the next section, we first elaborate on the theoretical foundations of the paper and then present the empirical data.

## Literature review

### Media framing in a pre-crisis context

Crisis life cycle theory [11, 12] suggests that crises follow a cycle or progress according to identifiable patterns. The pre-crisis stage is the first phase of the cycle characterized by uncertain disruption causes, the scope of impact, responsibility attribution, and intervention measures. In the pre-crisis context of COVID-19, such uncertainties include COVID-19 disease progression, availability of effective therapies for victims, patient outcomes (e.g., quality of life influenced by the disease or therapy), and more importantly, whether the situation warrants locking down the city despite the immense costs of restricting people’s freedom and economic stagnation [13, 14]. The more uncertain a situation is, the more intense the dilemmas faced by individuals or organizations, resulting in decisions that rely more on historical precedents and past habits [15]. Thus, the uncertainty during the COVID-19 pre-crisis period in China offers a rare opportunity to better understand the Chinese media’s structural decision-making processes in terms of public health content, framing, discourse, tone, and style during a period of great uncertainty.

Framing offers a “schemata of interpretation,” which helps individuals locate, perceive, identify, and order issues, events, or topics. Media framing is crucial during uncertain situations when routine reference frames or interpretive schema may not work to make sense of what is currently taking place [16, 17]. In democracies, mass media play three key roles in social life: 1) a free marketplace of ideas where various voices compete for public recognition without the intervention of the state; 2) public service broadcasting that fosters the understanding of issues and rational decision-making; and 3) the fourth estate [18]. Depending on the stage of a crisis, certain roles are more appropriate than others. During the initial crisis stage, given the unclear situation, in order not to disrupt the unity of the people and the morale of crisis managers, mass media’s marketplace and public service roles should focus on providing timely updates and broad information as the crisis unfolds, while its role as a watchdog is more appropriate at later stages in evaluating the performance of public officials during the crisis [17]. However, in the pre-crisis stage of COVID-19 in democratic contexts, news media’s roles of marketplace (e.g., discussion on COVID-19 restrictions and the effect on human right), public service (e.g., the timely reporting of developments of COVID-19), and watchdog (e.g., the origin of COVID-19) were all prominent [19–21] and manifested in news frames.

Crisis communication scholars have identified five key media frames during a crisis: human interest, economic consequences, conflict, morality, and responsibility [22, 23]. Human interest brings an individual’s experiences and humanistic concern to the presentations of crises. Economic consequences focus on economic harms that crises bring to individuals, groups, and organizations. Conflict presents the contradictions among different views of parties involved in crises. Morality is usually used indirectly to depict the moral, cultural, or religious backgrounds of crises. Responsibility identifies the individuals, groups, or organizations who should be blamed for causing crises. Several studies [17, 24, 25] found that human interest and economic consequences frames were mostly adopted in hurricane and flu crises coverage during the pre-crisis stage, while the conflict and responsibility frames were emphasized at later phases. However, when covering the COVID-19 crisis, apart from individual suffering and economic loss frames, media institutions in democratic countries also heavily emphasized the responsibility frame by painting China as being responsible for the outbreak of COVID-19 [26–28]. Thus, when a crisis is perceived as human-made with clear identification of the person or organization responsible, the media is more likely to engage in responsibility or conflict framing. For natural disasters, other frames dominate until clarity emerges later about responsibilities. Similar framing practices exist in Chinese media, but the structural characteristics of the Chinese media ecosystem also result in unique crisis news reporting strategies.

### Fragmented control of the Chinese media and its crisis communication practices

The Chinese media system is a combination of *Tiao* and *Kuai* [8, 29–32]. *Tiao* refers to the top-down “vertical” hierarchical power system in which central media institutions exert control over local media institutions. *Kuai*, or territorial management (*Shudi Guanli*) is a more horizontal system of governance that subordinates local media institutions to local government propaganda departments. The dual-track supervision model produces a uniquely Chinese media phenomenon where higher-level media institutions supervise local, regional governments by critically reporting on their activities to maintain social stability and a positive impression among the public [3, 8, 32]. Meanwhile, local media institutions similarly supervise other local governments instead of its affiliated local government by critically reporting on the activities of these governments, acting as “watchdogs for others’ houses” (*Yidi Jiandu*) [31]. The *Tiao* and *Kuai* dual-track arrangement sometimes results in conflicting instructions and expectations, opening autonomous spaces for social actors and institutions to act independently and engage in a potentially contentious activity despite the generally repressive style of governance in China, a phenomenon which Liberthal [29] and Mertha [30] termed “fragmented authoritarianism.”

According to the *Public Emergency Response Guidelines of China* [33, 34], the guiding principles of crisis reporting are timeliness, accuracy, and moderation. During emergencies when circumstances are unclear and potentially complicated, short and accurate messages should be timely sent, with follow-up reports made later. The moderation principle requires that crisis coverage should not cause social panic nor damage the image of the party and government [34]. To dilute the negative impact of severity frames in news reports, previous studies have found that the most frequent frames that co-occurred with the severity frame were party leadership and solution frames [35–37]. As Repnikova [3] argued, many crisis media reports employ subtle linguistic techniques, including the tone of coverage and the composition of frames. Media coverage of a crisis may not always be salient, explicit, and objective. Thus, examining whether a severity frame is presented and how it is conveyed are both necessary to differentiate between factual reports and implicit propaganda in China’s sensitive communication environment. The manner which crisis severity news reports are written may be closely related to the media organization’s position in the power hierarchy, crisis reporting strategies, and situational factors, which we will elaborate in the following paragraphs and relate them to our hypotheses.

In the vertical power hierarchy of *Tiao*, high-level media institutions, such as the central media, enjoy the exclusive power of information release and content formulation during the crisis. Hong [38] found that central media institutions reported the severity of SARS more frequently than the local-level media because the official protocol on crisis information release mandates that local media can only adapt officially vetted reports issued by senior media institutions in producing local news reports to ensure the accuracy and consistency of the crisis information across different channels [10, 35, 36]. Moreover, covering local governments’ incompetence and failures in managing crises allows senior media institutions to play a limited fourth estate role to resolve social contradictions and win over the public’s trust and support in a political environment anathema to press freedom [31, 32]. In the context of the pre-crisis phase of COVID-19 in China, high-level media institutions have a vested interest to intensify the weight of severity information in their published news articles to preserve their credibility with the public. Thus, we propose:

H1: High-level media institutions were more likely to emphasize the health severity frame than low-level media.

In terms of territorial media governance characterized by *Kuai*, the physical proximity of media institutions to the crisis epicenter may also adopt distinctive crisis reporting strategies. In the literature of news production, geographical proximity can explain differences in media behavior in this “horizontal” governance structure. Greater proximity is deemed more newsworthy, resulting in a larger number of detailed news reports [39]. In the context of public crisis, proximity can indicate “catastrophic potential” or “the degree of threat”, which may affect the risk perception of the public and media; it can correspondingly determine the priority of news agenda [39, 40]. Trope and Liberman [41] thus argued that proximity should be conceptualized as a multifaceted concept including both physical and social proximity. Thus, for China’s COVID-19 crisis, conceiving proximity as purely geographical is unsatisfactory because the effect of an epidemic is relatively less geographically localized than most ordinary natural disasters or man-made accidents [42]. Moreover, over 10 million people traveled into or moved out of Wuhan during the pre-crisis stage [43]. Hence, regions with a significant population of people who had been to Wuhan are clearly more socially proximate to Wuhan, and media institutions in these regions would be more concerned about the pre-crisis outbreaks in Wuhan regardless of geographical proximity. Social proximity thus could be a more salient consideration than geographical proximity for Chinese media institutions in determining the importance and prominence of Wuhan related news.

In addition to greater enthusiasm for reporting news in socially or culturally proximate areas, media institutions also favor the events taking place such proximate areas [44]. During the crisis of avian flu Hong Kong, Fung and colleagues [40] found that crisis reports from *South China Morning Post* are less likely to sensationalize the crisis and emphasize its dreadfulness than those from *New York Times*, which is socially distant from Hong Kong. A similar phenomenon of favorably reporting news from socially proximate regions also exists in China. To resist supervision from the central government and other regional media institutions, local governments and their affiliated institutions in proximate areas are increasingly forming local-level alliances to cover one another [9, 32, 35]. In the context of the COVID-19 crisis, this means provinces and cities nearer to Wuhan have greater incentive to collude with the Wuhan government to give less importance and prominence to negative severity news about Wuhan to ride out the crisis, hopefully without attracting too much attention from the central government, because these provinces and cities may suffer collateral damage if high-level investigations or “watchdogs for others’ houses” news reporting occurs. Thus, we propose:

H2: Media institutions socially proximate to Wuhan are less likely to emphasize the health severity framing than those that are socially distant.

The impact of media attributes on severity framing in news reports is not uniform and consistent and could be affected by specific coverage strategies. To prevent being made a scapegoat, low-level and socially-proximate media institutions can seize the opportunity to exercise editorial independence when there are inconsistencies in instructions from different higher-up institutions [32, 35, 45], facilitating independent crisis reporting through strategic cherry-picking of directions on news coverage by different higher-level institutions. Transgressions against the direction of any institution can be justified as obeying the instructions of another institution. For example, media institutions near a crisis incident can cherry-pick information to attribute the crisis to non-human factors (e.g., a rare virus that seldom exists before) instead of human negligence when reporting the crisis’s actual severity [46]. Such coping tactics of attribution transfer or shifting the focus not only exist in the discursive aspect but also in the physical aspect of article prominence [46, 47].

Many agenda-setting and framing studies have found that the selection of salient agendas is not a zero-sum game [48, 49]. Media institutions can accommodate different frames and moderate them by adjusting the prominence of a news article [47, 50]. Prominence signals the importance of the article through its positional placement relative to others. In media reports of the SARS epidemic, Xiong [46] found that the coverage of the crisis severity and political propaganda is almost equally prominent in quantity. However, reports about maintaining stability (e.g., fighting against rumors) generally appear on front pages, whereas reports about transmissibility was placed at the back. In contexts such as China, where media institutions often face the dilemma of having to meet both the government’s requirement of maintaining social stability and the standards of professional news reporting expected by the public, editorial independence in the choice of news frames is limited [8, 9]. To resolve the dilemma, a middle-ground compromise for junior media institutions socially proximate to Wuhan could be to vary the prominence of news reports by adjusting their positions, placing reports that may incur the ire of the government away from the front page. Given the lack of empirical evidence on how the positional placement of articles affect the selection and composition of news frames for different types of media, we thus ask:

RQ1: How can the article prominence moderate the associations between the level of media institutions (a) / social proximity (b) and the weight of severity framing?

The differentiation of media behaviors is not only the result of actively seeking editorial independence but also the passive response to changes in the external environment. An external factors that has an important influence on the decision-making of the public sector is the change in epidemic severity [51, 52]. The seriousness of the situation can increase the urgency to cope with external pressures. As stipulated in the *Public Emergency Response Guidelines of China* [33, 34], accurate and timely severity news reports are particularly important during a potential public health crisis because they signal what should be the appropriate level of public concern. Moreover, Wang [53] proposed the “popular pressure model” in political decision-making in China, suggesting that effective governance requires breaking from established norms to de-escalate conflicts between the people and the ruling party. Thus, the initial outbreaks of the then-novel coronavirus in Wuhan provide junior media organizations socially proximate to Wuhan a strong justification to break from established reporting protocol under the *Tiao* and *Kuai* arrangements to influence the weights of severity frames in articles. Failure to cope flexibly with the changing environment may result in huge conflicts between the population in the regions they serve and the ruling party. Given that few studies have explored the situational adjustments of media framing practices, we thus ask:

RQ2: How can the severity of the situation moderate the associations between the level of media institutions (a) / social proximity (b) and the weight of severity framing?

## Method and data

### Data collection

To examine news frames during the pre-crisis stage of the COVID-19 crisis in China, we employed content analysis on major newspaper reports from various provinces in China. We used two databases to collect media reports: *China Digital Library* and *Wise News*, which are the two most comprehensive databases of mainland Chinese newspapers. Using Fink’s [12] definition of the pre-crisis stage as the period where several clues of a potential crisis have emerged, we determined the pre-crisis phase of China’s COVID-19 epidemic as the period from 1 January 2020, when Wuhan’s local newspaper first confirmed the emergence of cases of pneumonia with unknown causes to 20 January 2020 with Dr. Zhong Nanshan, Chairman of the State Council’s Medical and Health Expert Group, announcing on China Central Television (CCTV) that the SARS-CoV-2 virus has been confirmed to be highly contagious and pose severe harm to personal health.

**Fig 1:**
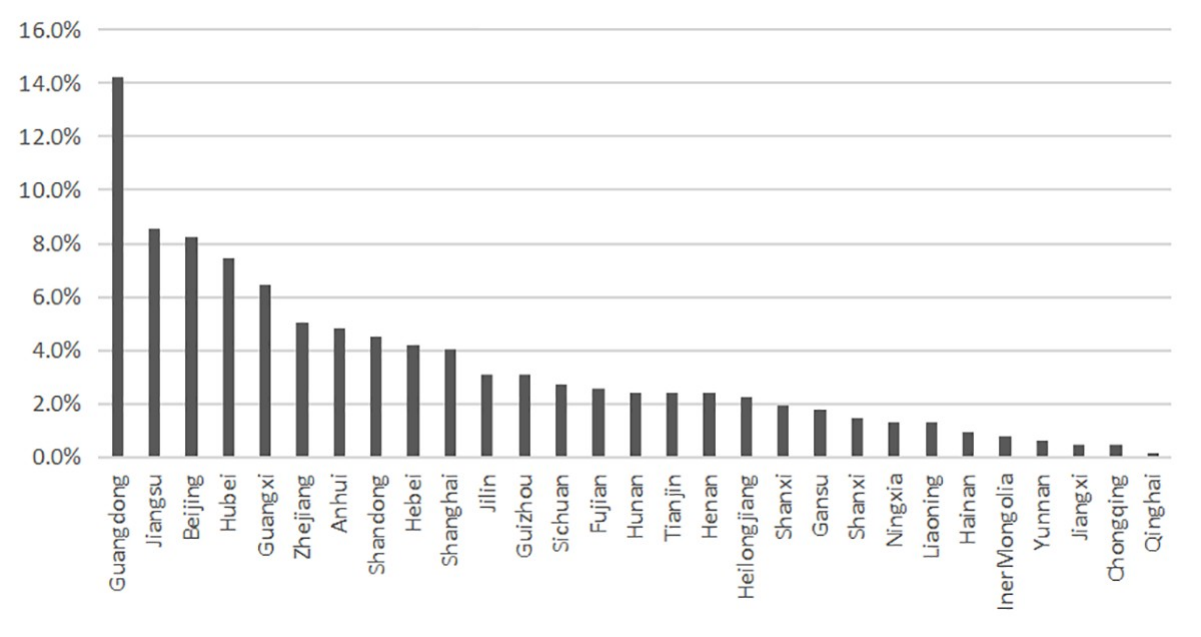
Distribution of news reports regarding COVID-19 during the pre-crisis stage by media institutions from different provinces.

Using keywords or phrases such as “Wuhan” (武汉) and “pneumonia” (肺炎), “Wuhan” (武汉) and “corona” (冠状), “Wuhan” (武汉) and “virus” (病毒), we retrieved 683 and 572 articles published by mainland media organizations from *China Digital Library* and *Wise News* respectively during this period on 5 March 2020. The initial searched of both databases returned many duplicates and reports unrelated to COVID-19, such as reports on influenza virus prevention in Wuhan. After deleting duplicated and irrelevant reports, the final dataset comprises 618 media reports from 152 different media organizations across 29 provinces, province-level municipalities, and autonomous regions (see Fig 1), among which Guangdong (14.2%), Jiangsu (8.6%), Beijing (8.3%), Hubei (7.4%) and Guangxi (6.5%) newspapers were the top 5 contributors.

### Coding process

#### The weight of a health severity frame as the dependent variable (DV)

We took studies on health crises [22–25], which have already identified several common themes introduced in the literature to establish the coding scheme. We also added three themes based on health crisis communication in the Chinese context. The first one is the governance frame, which is akin to what Luther and Zhou [36] named the ‘leadership’ frame, emphasizing what parties and governments have done to prevent the virus from spreading. The second frame relates to pacification, which aims to remove unnecessary panic by assuring people that those in charge are capable of managing the crisis [37, 45]. The last frame is suggested actions to take, relating to measures that citizens can perform to contain a health risk. Detailed definitions of coding frames and examples are shown in Table 1.

**Table 1:** The coding scheme of news frames in this study.

News frames	Operationalized definitions	Examples
Health severity	The adverse effects of COVID-19 on human health, including descriptions of disease symptoms, possibility of cure, difficulties of disease prevention, and transmissibility [22, 23].	Viral pneumonia is more terrible than AIDS because AIDS is mainly transmitted through sexual contact and blood.
Economic consequence	The economic stagnation caused by the outbreak of the virus, such as factory shutdowns, shortage of goods, and increased unemployment [22, 23].	Due to the unexplained pneumonia, many seafood products were stranded on the road near the seafood market and difficult to sell.
Human interest	The daily lives and experiences of ordinary citizens during an epidemic, such as their mental health and disruptions of daily routines [22, 23].	Ms. Wang, a merchant in the market, said that she is busy with business every day and doesn’t pay attention to the “viral pneumonia incident” until several confirmed cases appeared in her seafood market.
Morality	Judgement toward involved parties of COVID-19 crisis based on moral or religious tenets [22, 23].	At a press conference held by the Ministry of Health, Labour and Welfare of Japan, the spokesman said: Preventing secondary infection in China is the primary task, and nationality has nothing to do with this goal, so the patient’s nationality will not be announced.
Conflict	Involved parties of COVID-19 crisis express disagreeing views and pit against each other [22, 23].	A number of interviewed merchants showed less concern about the viral pneumonia. “There are hundreds of people in the market, and several people have flu, which is normal.”
Responsibility	Blaming people or institutions responsible for the emergence of the crisis [22, 23].	Without verification, some netizens publish and forward false information on the Internet, causing adverse social impact.
Governance	Emphasizing the measures, precautions, or regulations developed by the party and government to control the epidemic situation [23, 36].	The mainstream media reported in time and gave authoritative information, winning praise and trust from the audience.
Pacification	Giving people a sense of hope and confidence in defeating the epidemic, and encouraging the public to trust the government’s capacity, material reserves, and medical system [37, 45].	Among the confirmed cases, the proportion of severe cases is not much different from that of severe cases of common pneumonia, and all patients are well treated.
Suggested actions to take	Offering tips or actions that can prevent health threats, such as washing hands frequently, delaying travels, and paying attention to personal hygiene [23, 54].	People must pay attention to personal hygiene. When going to places with many people, they can wear masks, strengthen exercise, and enhance immunity when necessary.

**Fig 2:**
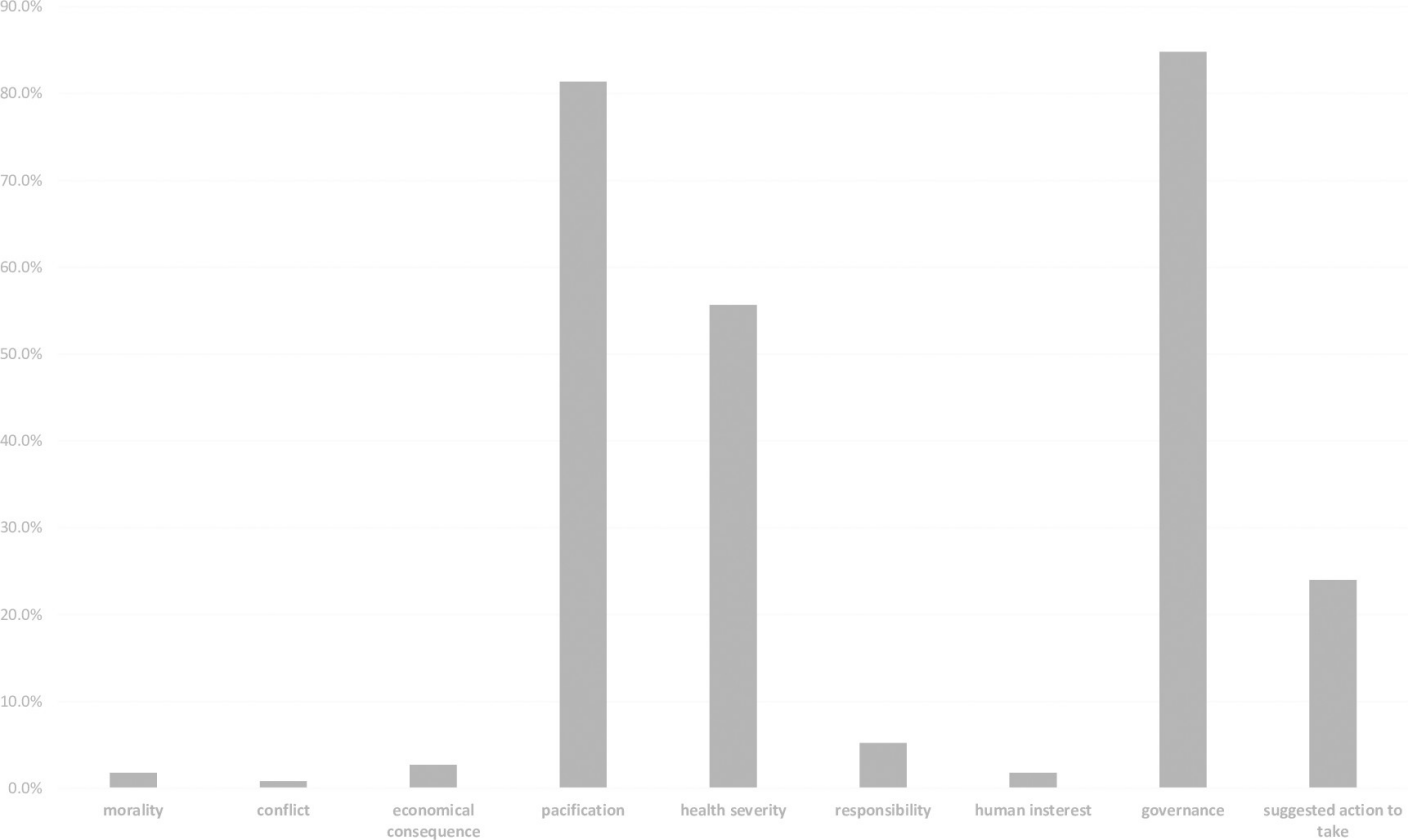
Frequencies of news frames in Chinese media reports on COIVD-19 during the pre-crisis stage.

After establishing the coding scheme, two trained graduate students majoring in communication coded the numbered news articles. Coders assessed the presence (1) or absence (0) of the above-listed news frames. After coders coded all news articles independently, we used the Random Number Generator (https://www.calculator.net/random-number-generator.html) to assist in selecting 62 articles (i.e., 10% of total news articles) to assess intercoder reliability between the coders. Krippendorff’s alpha [55] ranged from 0.76 to 0.88. The mean reliability for the seven items was 0.81, which was satisfactory. Fig 2 shows the frequencies of different news frames. As a story may contain multiple frames, the dependent variable is defined as the health severity frame’s weight in each report. For instance, if an article was composed of three key frames: health severity, economic consequence, and pacification, then the weight of the health severity frame would be 1/3 = .333. The average weight of the health severity frame in all media reports was 0.23.

### Independent variable (IV) and moderators

#### Level of a media institution

In China’s media system, a media organization’s level is tied to its affiliated government unit’s level [9]. For instance, because the *People’s Daily* is led by the State Propaganda Department, its level is central. In contrast, the *Wuhan Evening Newspaper* is led by the Wuhan Municipal Propaganda Department, so its level is municipal. From 1 January 2020 to 20 January 2020, the number of news articles from central media was relatively limited (n = 16). The control of epidemic situation and the dissemination of information still followed the principle of territorial management [56]. Provincial government agencies and their affiliated media organizations were the highest decision-making bodies and the front-line entities in leading and monitoring local public opinion. Hence, in this study, we defined central and provincial -level media as high-level media (40.0%), and municipal and county-level media as low-level media (60.0%).

#### Social proximity

This IV reflects the volume of human traffic between Wuhan and other provinces, including Hubei, Wuhan’s home province. To construct the variable, we collected commuting data from the *Baidu Migration* database (http://qianxi.baidu.com/) developed by Baidu Group. Through the Application Programming Interfaces (APIs) of airports, railway stations, expressways, and docks across the country, *Baidu Migration* offers daily population inflow and outflow statistics in over 100 major cities across the country. Using *Baidu Migration*, we obtained the destination provinces and proportions of Wuhan’s population outflow from 1 January 2020 to 20 January. 2020. The social proximity between Wuhan and the province where a media institution is located is determined by the average proportion of Wuhan’s population flowing into the province from the 1st to the 20th. The higher the proportion of Wuhan’s population flow to a province, the higher the social proximity of that province to Wuhan. On average, provinces received 3.81% of the outflow population from Wuhan during the period.

#### Prominence of a news article

This moderator reflects the extent to which a media report is considered important [50]. Apart from frame choice, adjustment of report prominence is another method to vary the balance between information control and information transparency [46, 47]. We measured this variable by evaluating the page an article (M = 7.79, SD = 5.11) appeared in. A smaller page number indicates higher prominence.

#### Severity of the situation

Crisis severity can affect organizations’ response strategies at specific time points. We measured severity using the official numbers of the sum of infected and suspected cases on a given day. Wuhan’s Municipal Health Commission (http://wjw.wuhan.gov.cn/) provided updates from 31 December 2019 to 20 January 2020, varying between 27 and 778 cases with an average of 294.4. A higher of reported infected and suspected cases reflects a more severe situation.

### Data analysis

We constructed a moderation model by regarding the level of a media institution and the social proximity of a media institution to Wuhan as IVs, the severity of the situation and article prominence as moderators, and the weight of a health severity frame as DV. We used the day as the unit of analysis when performing the regression analysis. To ensure that there’s a zero value for meaningful interpretation, we mean-centered the continuous IV and moderators when testing the hypotheses and exploring the questions using R. Given that the DV is a proportional variable, we employed a generalized linear model (GLM) with binomial errors when running the statistical analysis. Moreover, the moderator, “the severity of the situation,” is an environmental factor collected at a different level compared to other IVs and moderator. Because the data are nested by article publication date, we employed a multi-level regression analysis when performing the cross-level interaction analysis between the severity of the situation and IVs.

## Results

We employed GLM with binomial errors to explore the impact of the level of media institutions (H1) and social proximity (H2) on the weight of a health severity frame in a news article. As shown in Model 1 in Table 2, as compared to the low-level media, high-level media did not show a significant difference (B = 0.15, SE = 0.19, p>0.05) in weighting the severity frame; thus, we rejected H1. Conversely, the coefficient indicated that the more proximate to Wuhan a media institution was, the more a health severity frame would be diluted in a news article (B = -0.01, SE = 0.01, p < .05), supporting H2. We further conducted a qualitative analysis to explore what frames have been used to downplay the weight of health severity frame. We found that the themes most frequently juxtaposed with the severity frame are pacification (90.6%) and governance (76.6%).

**Table 2:** Regression and moderation analyses on examining the effect of media attributes on the weight of severity frame.

	Model 1	Model 2	Model 3
	B (SE)	B (SE)	B (SE)
Intercept	-1.21 (0.12)***	-1.29 (0.13)***	-1.42 (0.17)***
***Fixed-effect model***			
Level of media (LOM) (reference: low-level media)	0.15 (0.19)	0.16 (0.20)	0.10 (0.20)
Social proximity (SP) (multiplying by 100)	-0.01 (0.01)*	-0.02 (0.02)*	-0.06 (0.03)**
Page		0.08 (0.02)**	0.08 (0.02)***
LOM*Page (reference: low-level media)		-0.13 (0.04)**	-0.14 (0.04)**
SP*Page		0.00 (0.00)*	0.00 (0.00)*
***Cross-level interaction***			
Severity of the situation (SOS)			0.08 (0.06)
LOM*SOS (reference: low-level media)			0.03 (0.08)
SP*SOS			0.01 (0.01)*
**N**	618	618	618
**Df**	617	617	617
**Pseudo R**^**2**^	0.01	0.03	0.32
**Akike Information Criteria (AIC)**	400.21	390.74	197.0

Note.

* *p* < .05.

** *p* < .01.

*** *p* < .001.

B: unstandardized coefficient.

**Fig 3:**
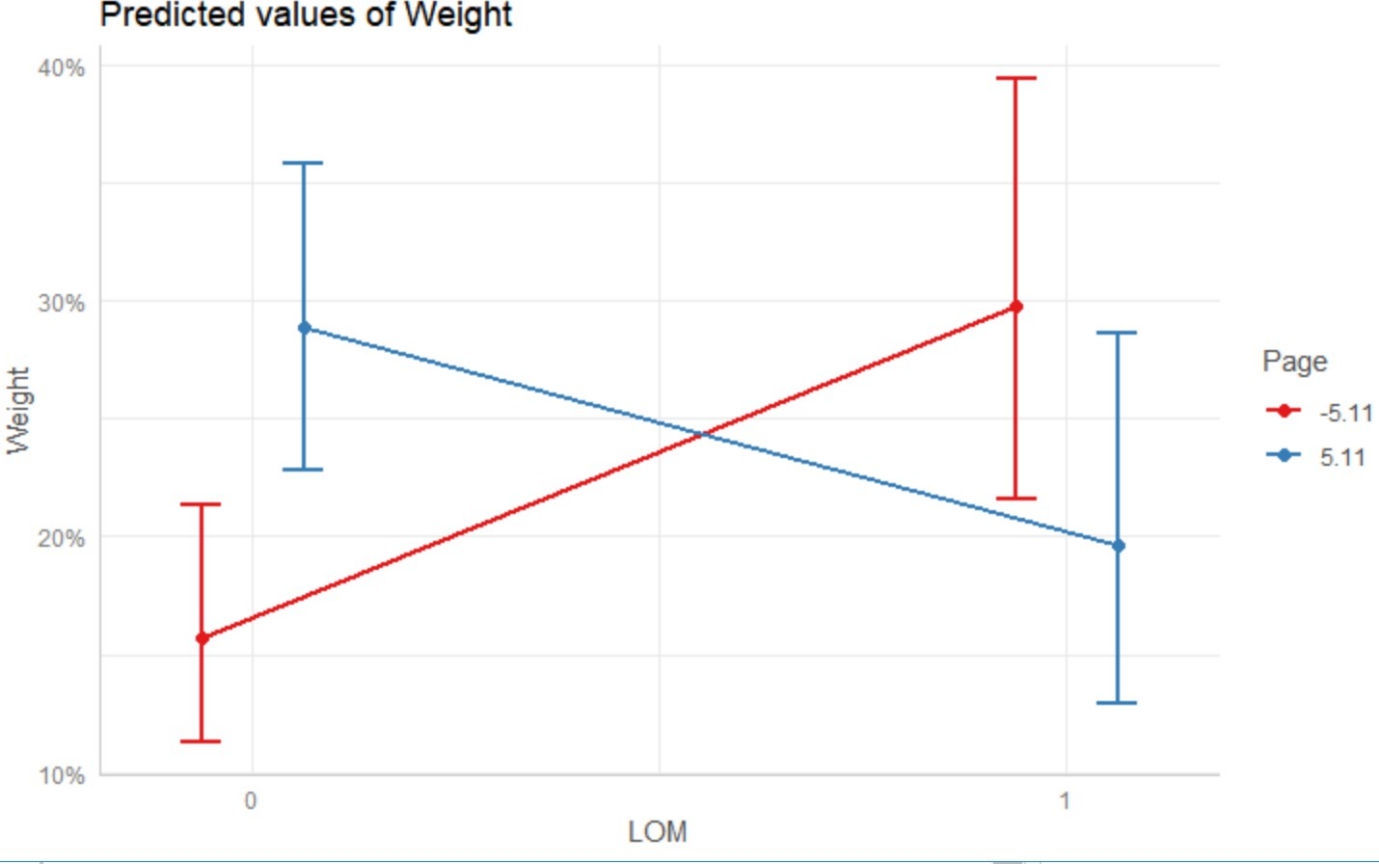
The interaction effect between the level of media (LOM) and page. Conditions for the moderator are the plus/minus one standard deviation from the mean.

**Fig 4:**
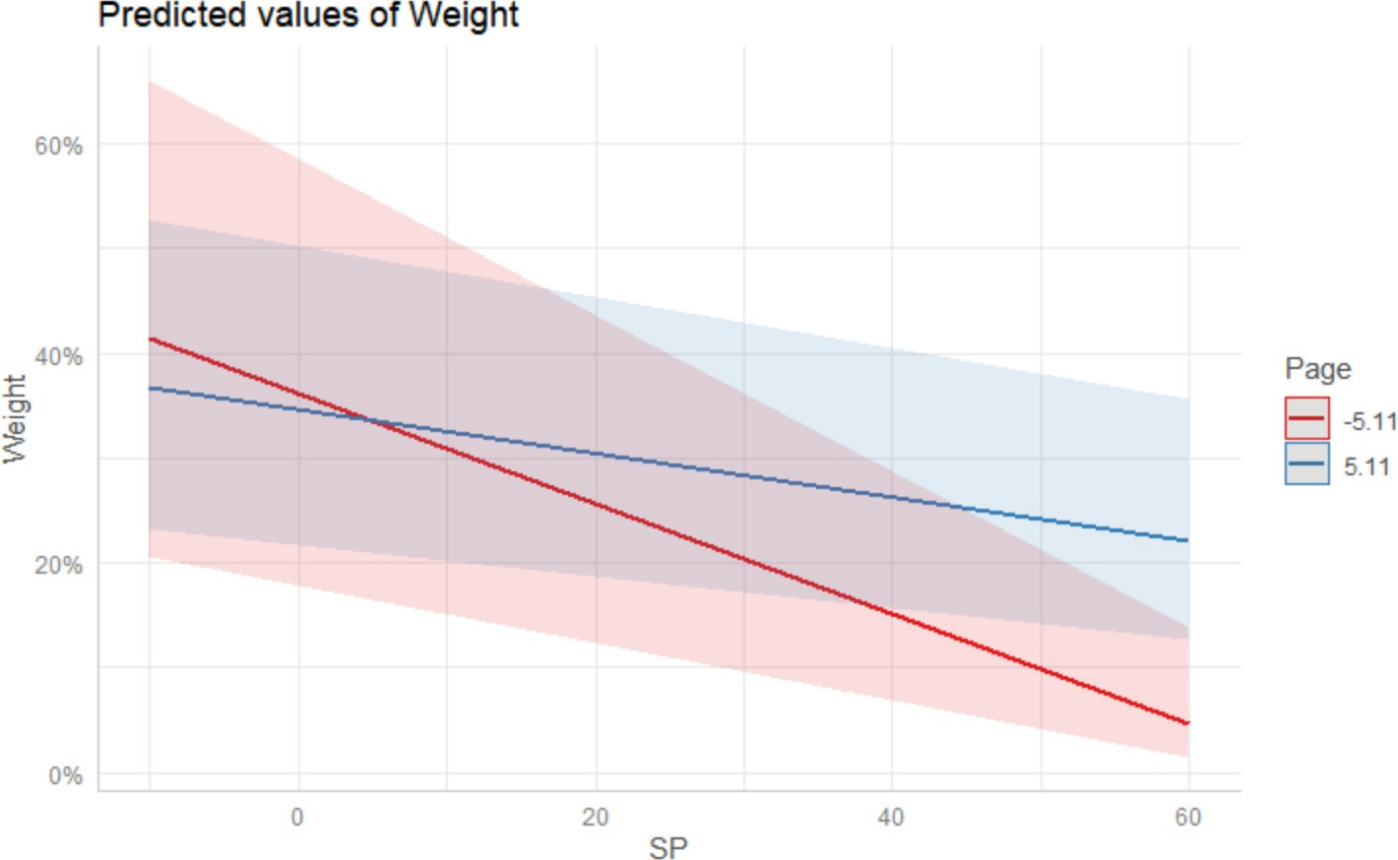
The interaction effect between the page position and Social Proximity (SP). Conditions for the moderator are the plus/minus one standard deviation from the mean.

To answer RQ 1, we ran the interaction analysis taking article prominence as the moderator. As shown in Model 2, article prominence can significantly moderate the two main effects. Specifically, the page position diminished the positive association between the level of media institutions and the weight of a health severity frame (B = -0.13, SE = 0.14, p < 0.01), suggesting that low-level media covered the severity frame more enthusiastically than the high-level counterparts as the page number increased (see Fig 3). Moreover, the page position (B = 0.00, SE = 0.00, p < 0.05) negatively moderated the negative relation between social proximity and the weight of a health severity. When the page number increased, the gap between socially-proximate and socially-distant media’s enthusiasm to cover the epidemic severity narrowed (see Fig 4).

**Fig 5:**
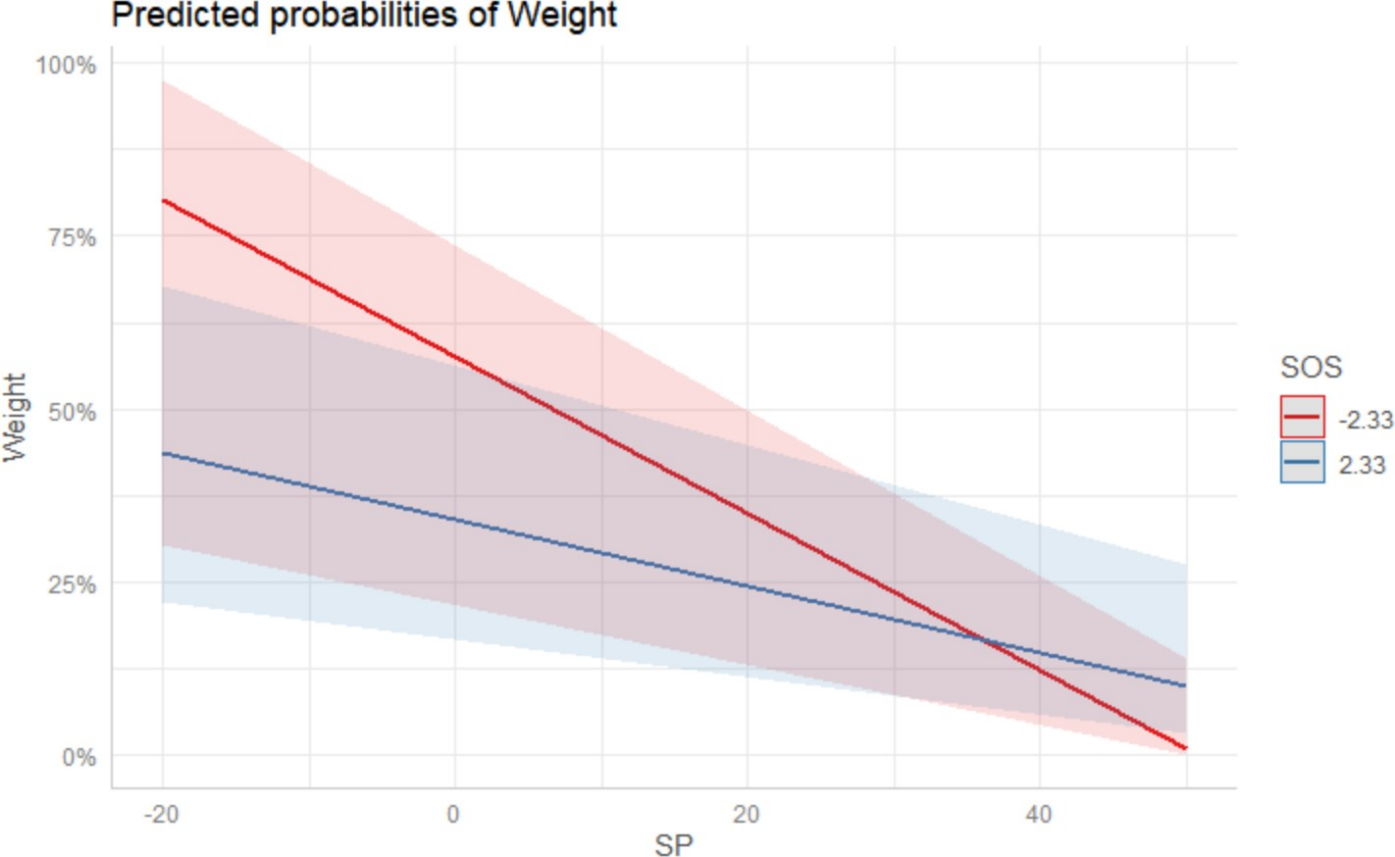
The interaction effect between the severity of the situation (SOS) and social proximity (SP). Conditions for the moderator are the plus/minus one standard deviation from the mean.

Finally, we ran a multilevel interaction analysis to explore whether the severity of the situation can moderate the main effect model (RQ2). The result in Model 3 revealed that the crisis severity (B = 0.01, SE = 0.01, p < 0.05) negatively moderated the negative association between social proximity and the weight of a health severity frame. The advantage of socially-distant media on emphasizing the severity frame over the socially-proximate narrowed as the situation became serious (see Fig 5). However, the crisis severity (B = 0.03, SE = 0.08, p > 0.05) did not significantly affect the association between the level of media institutions and the weight of the severity frame.

## Discussion

Akin to the crisis framing practices in western democratic countries, mass media in China has also acted as an information gatekeeper and high-quality information provider in the pre-crisis stage in China [22–25]. Our findings indicate that during the pre-crisis period, Chinese media focused on the communication of governmental measures, pacification of public sentiments, timely severity information updates, and the dissemination of health knowledge. However, the difference with democratic countries was that coverage generally communicated the seriousness of the epidemic implicitly: governance and pacification frames were frequently used to dilute the weight of the severity frame, which Brady [35] called “framing the problem with hope.” In addition, unlike media reports in the pre-crisis stage of COVID-19 in a democratic context [19–21], frames about economic losses, dissenting voices, or people’s daily life were limited, which is not unexpected as they would have undermined the foundation of the CCP’s governance legitimacy [34]. In this sense, moderated reporting during the pre-crisis stage appeared to serve the twin goals of informing the public of the truth and preserve the legitimacy of the ruling party at the same time. However, not all Chinese media institutions closely adhere to the principles of timeliness, accuracy, and moderation [33, 34]. Extending previous research on fragmented authoritarianism in China, we not only illustrate that the differentiation of media behaviors is due to the availability of opportunities and tensions within institutions, but also contribute to demonstrating the nuances of where, how, and when the distinctive media practices take place.

As Liberthal [29] and Mertha [30] argued, China’s gigantic size and heterogeneity of interests enable factionalism and localism to thrive, accounting for the fragmentation of news reporting behavior despite the authoritarian nature of governance. Echoing Xia and Yuan’s [31] insights, autonomous farming practices are more likely to appear in China’s horizontal axis of the media system. Our findings that media institutions socially proximate to Wuhan are more enthusiastic than the distant counterpart to downplay the severity of the epidemic situation and portray an optimistic vision that everything is under control provide support for Zhou’s [57] contention that neighboring institutions tend to collude. As for the vertical axis of power structure, high-level media organizations did not clearly discharge their social responsibility as expected. As shown in Fig 3, although high-level media covered the crisis severity more saliently in front pages, it is the low-level media who contributed more to the transparent dissemination of the crisis when the article prominence reduced.

In this sense, the power status and credibility of high-level media do not necessarily manifest in the large number of crisis news reports that adopt a severity frame [36, 38], but manifest in how they present an article that details the crisis severity (i.e. a prominent page position). For low-level media, they only enjoy the relative autonomy of placement of news frames in inconspicuous page positions. Xia and Yuan [31] argued that higher-level media institutions in China often scapegoat lower-level institutions in the name of supervision to absolve themselves of potential blame for earlier news reporting mismanagement. Thus, lower-level media institutions generally err on the side of caution and adjust the article prominence to avoid offending superior institutions. Similarly, as shown in Fig 4, during COVID-19 pre-crisis stage, by reducing the prominence of truthful news reports about the severity of the impending epidemic, media institutions socially proximate to Wuhan can shift public focus and reduce anxiety while simultaneously eluding accountability and claiming transparency in managing the situation should the situation further deteriorate. Hence, for a low-level media institution socially proximate to the crisis epicenter in China, it is often necessary to sacrifice the positional placement in exchange of the factual and transparent crisis reporting.

Our research also found that the increase of external pressure can also cause the differentiation of media behavior, especially in the horizontal dimension of social proximity. Fig 5 illustrates SOS’s contrasting effect on deciding the weight of a severity frame among media with different social distances from Wuhan. As the situation became increasingly severe, socially-proximate media’s enthusiasm to report severity information surpassed the socially-distant. Consistent with the “popular pressure model” [53], when the perceived external severity was strong enough, going against the usual procedure of media and casting aside the interests of alliances is necessary because failing to do so may increase culpability in later investigations. In this sense, under the *Tiao-Kuai* dual-track media management system, the increasing seriousness of the situation, and public pressure is a double-edged sword. On the one hand, it can make the socially-proximate media actively perform their public service duties; on the other hand, it also makes media organizations socially distant act cautiously by depriving them of political opportunities to act the role of “watchdog in others’ houses” [8, 31–32]. If the collusion between regions and the inconsistency between superior and subordinate regulations are prerequisites for the editorial independence of low-level media in socially remote areas [3, 4, 8, 31, 32], then the disintegration of collusion and the emphasis on the consistency of information release across levels caused by the deterioration of the pre-crisis COVID-19 situation shrank the available autonomous spaces that socially-distant media could have enjoyed to conduct independent reporting.

Overall, our findings illustrate when, where and how spaces for independent news reporting open up and how they are capitalized on in a fragmented media system. Such transience and conditionality of spaces for independent news reporting are rooted in China’s unique news system and political culture. Inheriting the Soviet management style and values [9], the Chinese media system eschews freedom of the press and acts as a tool to aid governance. During a crisis, unfettered news reporting can complicate crisis management efforts by amplifying fear and distrust of organizations and individuals managing the crisis [58]. An unregulated press also results in information inconsistency that confuses the public, negative reporting affecting the morale of crisis managers, and emotionally-charged interviews of victims that incite public discontent, all of which are obstacles to crisis handling [13, 59]. Also, the ruling CCP government bears the brunt of social pressure in a Chinese political culture that lacks institutional checks and balances with few political release valves to ease pent-up dissatisfaction with the government [53]. The government is regarded as solely responsible in crises [58, 60], which makes many crisis communication strategies based on attribution transfer [11], which work well in liberal democracies, difficult to apply in China’s political context because there are few other political actors. Based on “time” (*shi*) and “degree” (*du*), different media institutions in China can acquire autonomous spaces to report truthful news, given that it is also an act of political self-preservation for the government.

## Limitations and implications

There are several limitations to this study. Firstly, given that the vast majority of our sample is state-run media, we lack a sample size of sufficient power for statistical analysis on commercial media news reporting during the pre-crisis stage. Future studies can investigate whether the ownership of media institutions also moderates reporting strategies during a public health crisis. Secondly, we did not examine the media organizations’ crisis coverage strategy on social media for comparison with print coverage [61], given that very few organizations were authorized to provide epidemic-related updates on social media during the pre-crisis stage. Future studies can examine if there are differences in offline and online mass media coverage of public crises in China.

Despite the limitations, our study shows that in the Chinese context, mass media continue to play a vital role in disseminating early warnings about possible outbreaks of novel or deadly diseases. Based on the time, degree, as well as attributes of media institutions, the actual severity of the situation can be discerned from media reports. Implicit codes or signs such as the level of media and the prominence of a news report are important for early detection of the emergence of future public health crises in an authoritarian context, where strong information control often means that official confirmation comes much later, resulting in loss of precious time to take mitigation measures. Public health news reporting in authoritarian mass media can provide critical information about ground realities if one knows when and where the independent news reports are and how to read the signs.

## Conclusion

In conclusion, our findings illustrate that news reports in China during an emerging public health crisis cannot be interpreted at face value. Although Chinese news generally media strengthened the coverage of the health severity frame as the crisis deepened, China’s highly fragmented media system resulted in differentiated moderation of news reporting, with social proximity and media institutional levels influencing the health severity framing of news reports depending on the article prominence and the epidemic severity. Although our findings demonstrate that the fragmented nature of the Chinese media system has allowed mass media to creatively work within severe limitations on editorial freedom to alert the public to an emerging public health crisis, the same fragmented nature of the media system unfortunately also limits its effectiveness in conveying crisis information in a timely manner.

## References

[1] Wicke P, Bolognesi MM. Framing COVID-19: How we conceptualize and discuss the pandemic on Twitter. PLoS One. 2020;15(9). 10.1371/journal.pone.0240010 32997720 PMC7526906

[2] Gilbert G, Sutherland M. The paradox of managing autonomy and control: an exploratory study. South African Journal of Business Management. 2013; 44(1): 1–14.

[3] Repnikova M. Media politics in China improvising power under authoritarianism. Cambridge: Cambridge University Press, 2017.

[4] Roberts ME. Censored: Distraction and Diversion Inside China’s Great Firewall. Princeton: Princeton University Press, 2018.

[5] Veil SR, Yang A. Media manipulation in the Sanlu milk contamination crisis. Public Relat. Rev. 2012; 38(5): 935–937.

[6] Li Y, Long Q. Reconstructing hegemony in the context of new media: The Weibo account of People’s Daily and its communicational adaptation (2012–2014). Communication & Society. 2017; 39: 157–187.

[7] Schlæger J, Jiang M. Official microblogging and social management by local governments in China. China Information. 2014; 28(2): 189–213.

[8] Xi Y, Ng A. Implied truth, complementary media practices, and successful atomized activism in China. Global Media and China. 2020; 5(3): 275–293.

[9] Zhao Y. From commercialization to conglomeration: The transformation of the Chinese press within the orbit of the party state. Journal of Communication. 2000; 50(2): 3–26.

[10] Zhao Y. Communication in China: Political economy, power and conflict. Lanham: Rowman & Littlefield, 2008.

[11] Coombs WT. Ongoing Crisis Communication: Planning, Managing and Resounding. London: Sage, 2012.

[12] Fink S. Crisis Management: Planning for the Inevitable. New York: AMACOM, 1986.

[13] Koffman J, Gross J, Etkind SN, Selman L. Uncertainty and COVID-19: how are we to respond?. J R Soc Med. 2020;113(6):211–216. 10.1177/0141076820930665 32521198 PMC7439590

[14] Abbasi K. COVID-19: fail to prepare, prepare to fail. J R Soc Med 2020; 113: 131. 10.1177/0141076820918796 32286118 PMC7160791

[15] Kochenderfer MJ. Decision making under uncertainty: Theory and application. Cambridge: MIT Press, 2015.

[16] Cortiñas-Rovira S, Pont-Sorribes C, Alonso-Marcos F. Simulating and dissimulating news: Spanish media coverage of the Swine Flu Virus. J. Cont. Crisis Manag. 2014; 23(3): 159–168.

[17] Pan P, Meng J. Media frames across stages of health crisis: A crisis management approach to news coverage of flu pandemic. J. Cont. Crisis Manag. 2016; 24(2): 95–106.

[18] Voltmer K. Mass media and political communication in new democracies. Sussex: Psychology Press, 2006.

[19] Poirier W, Ouellet C, Rancourt M-A, Béchard J, Dufresne Y. (Un)Covering the COVID-19 Pandemic: Framing Analysis of the Crisis in Canada. Canadian Journal of Political Science. 2020;53(2):365–371.

[20] Jo W, Chang D. Political Consequences of COVID-19 and Media Framing in South Korea. Frontiers in Public Health. 2020;8. 10.3389/fpubh.2020.00425 32974260 PMC7481441

[21] Ogbodo JN, Onwe EC, Chukwu J, et al. Communicating health crisis: a content analysis of global media framing of COVID-19. Health Promot Perspect. 2020;10(3):257–269. 10.34172/hpp.2020.40 32802763 PMC7420175

[22] An S, Gower KK. How do the news media frame crises? A content analysis of crisis news coverage. Public Relat. Rev. 2009; 35(2): 107–112.

[23] Dan V, Raupp J. A systematic review of frames in news reporting of health risks: Characteristics, construct consistency vs. name diversity, and the relationship of frames to framing functions. Health Risk Soc. 20(5–6); 2018:1–24.

[24] Vasterman PL, Ruigrok N. Pandemic alarm in the dutch media: media coverage of the 2009 influenza a (H1N1) pandemic and the role of the expert sources. Eur. Journal. Commun. 2013; 28(4): 436–453.

[25] Ungar S. Global Bird Flu Communication: Hot Crisis and Media Reassurance. Sci. Commun. 2008; 29(4): 472–497.

[26] Jia W, Lu F. US media’s coverage of China’s handling of COVID-19: Playing the role of the fourth branch of government or the fourth estate? Global Media and China. 2021;6(1):8–23.

[27] Bolsen T, Palm R, Kingsland JT. Framing the Origins of COVID-19. Sci. Commun. 2020;42(5):562–585. 10.1177/1075547020953603 PMC7484600 38603006

[28] Thomas T, Wilson A, Tonkin E, Miller ER, Ward PR. How the Media Places Responsibility for the COVID-19 Pandemic—An Australian Media Analysis. Frontiers in Public Health. 2020;8. 10.3389/fpubh.2020.00483 32974266 PMC7472525

[29] Lieberthal K. Governing China: From revolution through reform. New York: W.W. Norton, 2004.

[30] Mertha A. "Fragmented authoritarianism 2.0": Political pluralization in the Chinese policy process. The China Quarterly. 2009; 200: 995–1012.

[31] Xia Q, Yuan G. The opportunity structure of "state" division, network control and dissemination of conflicting Topics. Open Times. 2014; 1: 190–208.

[32] Guan B, Xia Y, Cheng G. Power Structure and Media Autonomy in China: The Case of Southern Weekend. J. Contemp. China. 2016;26(104):233–248.

[33] National Health and Family Planning Commission of the People’s Republic of China (NHFPC). The Ministry of Health’s Information Dissemination Plan on Statutory Infectious Diseases and Public Health Emergencies. 2006. Available at http://www.nhc.gov.cn/zwgkzt/wsbysj/200902/39121.shtml (assessed 15 March 2020).

[34] Yang Z. Various dilemmas on news communication. The State Council Information Office of the People’s Republic of China. 2010. Retrieved from http://www.scio.gov.cn/wlcb/llyj/Document/827757/827757.htm

[35] Brady AM. Marketing dictatorship: Propaganda and thought work in contemporary China. Lanham: Rowman & Littlefield, 2008.

[36] Luther CA, Zhou X. Within the Boundaries of Politics: News Framing of Sars in China and the United States. J. Mass Commun. Q. 2005;82(4):857–872.

[37] Han EL. Microblogging memories: Weibo and collective remembering in contemporary China. London: Palgrave Macmillan, 2015.

[38] Hong T. Information control in time of crisis: the framing of SARS in China-based newspapers and Internet sources. Cyberpsychol Behav. 2007; 10(5): 696–699. 10.1089/cpb.2007.9968 17927538

[39] Kwon KH, Chadha M, Pellizzaro K. Proximity and terrorism news in social media: A construal-level theoretical approach to networked framing of terrorism in Twitter. Mass Commun. Soc. 2017; 20(6): 869–894.

[40] Fung TK, Namkoong K, Brossard D. Media, Social Proximity, and Risk: A Comparative Analysis of Newspaper Coverage of Avian Flu in Hong Kong and in the United States. J. Health Commun. 2011;16(8):889–907. 10.1080/10810730.2011.561913 21590569

[41] Trope Y, Liberman N. Construal-level theory of psychological distance. Psycho. Rev. 2010;117(2): 440–463. 10.1037/a0018963 PMC3152826 20438233

[42] Feng Y, Li Q, Tong X, Wang R, Zhai S, Gao C, et al. Spatiotemporal spread pattern of the COVID-19 cases in China. PLoS One. 2020; 15(12):e0244351. 10.1371/journal.pone.0244351 33382758 PMC7775067

[43] Sun Y, Meng L, Chu Y. How many people left Wuhan before the Spring Festival in 2020?. 2020. Available at https://www.jiemian.com/article/3903117_qq.html (assessed on 3 March 2020).

[44] Kwon KH, Chadha M, Wang F. Proximity and networked news public: structural topic modeling of global twitter conversations about the 2017 quebec mosque shooting. Int. J. Commun. 2019; 13: 2652–2675.

[45] Lei YW. The Contentious Public Sphere: Law, Media, and Authoritarian Rule in China. Princeton University Press, 2018.

[46] Xiong W. Research on the idea, paradigm and countermeasures of the report of the public health events in China. Master thesis in Soochow University, China. 2010.

[47] Cheng Q, Chen F, Yip PS. The foxconn suicides and their media prominence: is the werther effect applicable in china?. BMC Public Health. 2011; 11: 841. 10.1186/1471-2458-11-841 22044598 PMC3233608

[48] Althaus SL, Tewksbury D. Agenda setting and the "new" News: Patterns of issue importance among readers of the paper and online versions of the New York Times. Commun. Res. 2002; 29(2): 180–207.

[49] Myhre SL, Saphir MN, Flora JA, Howard KA, Gonzalez EM. Alcohol coverage in California newspapers: Frequency, prominence, and framing. J. of Public Health Policy. 2002; 23(2): 172–190. 12108117

[50] Lee S. International public relations as a predictor of prominence of us news coverage. Public Relat. Rev. 2007; 33(2): 158–165.

[51] Vai B, Cazzetta S, Ghiglino D, et al. Risk Perception and Media in Shaping Protective Behaviors: Insights From the Early Phase of COVID-19 Italian Outbreak. Front. Psycho. 2020;11. 10.3389/fpsyg.2020.563426 33250809 PMC7674945

[52] Zhang W, Xi Y, Chen A. Why do replies appear? A multi-level event history analysis of online policy discussions. New Media Soc. 2020;22(8):1484–1504.

[53] Wang S. Changing models of China’s policy agenda setting. Modern China. 2008; 34(1): 56–87.

[54] Li Z, Hsu M. News framing of haze in People’s Daily in China (2011–2017). Mass Media Research. 2020; 142: 59–109.

[55] Krippendorff K. Reliability in content analysis: Some common misconceptions and recommendations. Hum. Commun. Res. 2004; 30(3): 411–433.

[56] See China Central Television’s (CCTV) interview with Wuhan Mayor Zhou Xianwang on 27 January 2020 at https://m.weibo.cn/status/IrCCD6tAU?fromvsogou=1#&video. Zhou made a detailed review and explanation of the epidemic management and information dissemination in the early stage of the epidemic.

[57] Zhou X. The institutional logic of collusion among local governments in China. Mod. China. 2010; 36(1): 47–78.

[58] Huang YHC, Wu F, Cheng Y. Crisis communication in context: cultural and political influences underpinning chinese public relations practice. Public Relat. Rev. 2016; 201–213. 10.1016/j.pubrev.2015.11.015 32288052 PMC7126396

[59] Tian Z. Contemporary disaster news research in China. PhD Thesis, Fudan University, China. 2005.

[60] Fu K, Zhou L, Zhang Q, Chan Y, Burkhart F. Newspaper coverage of emergency response and government responsibility in domestic natural disasters: China-US and within-China comparisons. Health Risk Soc. 2012; 14(1): 71–85.

[61] Van der Meer TG, Verhoeven P. Public framing organizational crisis situations: Social media versus news media. Public Relat. Rev. 2013; 39(3): 229–231.

